# Elimination of Teratogenic Human Induced Pluripotent Stem Cells by Bee Venom via Calcium-Calpain Pathway

**DOI:** 10.3390/ijms21093265

**Published:** 2020-05-05

**Authors:** Aeyung Kim, Seo-Young Lee, Bu-Yeo Kim, Sun-Ku Chung

**Affiliations:** 1 Clinical Medicine Division, Korea Institute of Oriental Medicine, Daejeon 34054, Korea; 2 Herbal Medicine Research Division, Korea Institute of Oriental Medicine, Daejeon 34054, Korea; 09seoyoung03@kiom.re.kr (S.-Y.L.); buykim@kiom.re.kr (B.-Y.K.)

**Keywords:** bee venom, induced pluripotent stem cells, apoptosis, necroptosis, calcium, calpain, reactive oxygen species, focal adhesion, teratoma

## Abstract

Induced pluripotent stem cells (iPSCs) are regarded as a promising option for cell-based regenerative medicine. To obtain safe and efficient iPSC-based cell products, it is necessary to selectively eliminate the residual iPSCs prior to in vivo implantation due to the risk of teratoma formation. Bee venom (BV) has long been used in traditional Chinese medicine to treat inflammatory diseases and relieve pain, and has been shown to exhibit anti-cancer, anti-mutagenic, anti-nociceptive, and radioprotective activities. However, the potential benefits of BV in iPSC therapy, particularly its anti-teratoma activity, have not been examined. In this study, we found that BV selectively induced cell death in iPSCs, but not in iPSC-derived differentiated cells (iPSCs-Diff). BV rapidly disrupted cell membrane integrity and focal adhesions, followed by induction of apoptosis and necroptosis in iPSCs. We also found that BV remarkably enhanced intracellular calcium levels, calpain activation, and reactive oxygen speciesgeneration in iPSCs. BV treatment before in ovo grafting efficiently prevented iPSC-derived teratoma formation. In contrast, no DNA damage was observed in iPSCs-Diff following BV treatment, further demonstrating the safety of BV for use with iPSCs-Diff. Taken together, these findings show that BV has potent anti-teratoma activity by eliminating residual iPSCs, and can be used for the development of effective and safe iPSC-based cell therapies.

## 1. Introduction

Human pluripotent stem cells including human embryonic stem cells and human induced pluripotent stem cells (iPSCs) are capable of indefinite self-renewal and pluripotent differentiation, which provide considerable usefulness in potential cell-based therapies [1,2]. Clinical use of embryonic stem cells has been limited due to ethical concerns regarding the use of embryos [3]. In contrast, iPSCs can be generated from various adult somatic cells via the introduction of reprogramming factors, thereby avoiding any ethical concerns [4]. Using various in vitro differentiation technologies, iPSCs can be differentiated into a wide variety of tissues for use in numerous cell-based regenerative treatments, including treatment of damaged tissues or degenerative diseases. In addition, iPSCs can be applied as a valuable resource for disease modeling and drug screening [2,5,6]. However, there remain serious safety concerns for iPSC therapy, as a subset of undifferentiated cells often remain within the differentiated cell mixture; these can form benign teratomas or aggressive teratocarcinomas after in vivo injection [7,8]. Therefore, complete elimination of iPSCs in the final cell products without compromising their viability, efficacy, and functional properties is necessary for the success of any iPSC-based cell therapy. In this regard, several strategies have been used to remove residual pluripotent stem cells from differentiated cell cultures, including small molecules, cytotoxic antibodies, and chemical inhibitors [9,10,11,12,13,14]; cell sorting using pluripotent stem cell-specific antibodies [15]; and the introduction of suicide genes into pluripotent stem cells [16]. However, each of these strategies has shown limitations in terms of specificity, safety, efficacy, and throughput. Therefore, it is necessary to develop novel agents or alternative strategies that can efficiently and selectively eliminate pluripotent stem cells while maintaining the viability of the differentiated cell population.

Bee venom (BV) is a bitter, colorless liquid that is synthesized and secreted by a gland located in the abdominal cavity of the honeybee. BV is composed of a complex mixture of biologically active peptides, including melittin (MLT, 40–50% of dry BV), apamin (2–3%), mast cell degranulating-peptide 401 (2–3%), and adolapin (1%); enzymes such as phospholipase A2 (PLA2, 10–13%) and hyaluronidase (1%); biologically active amines such as histamine (0.2–2%) and dopamine (0.2–1%); and non-peptide components, such as lipids, carbohydrates, and free amino acids [17]. In traditional Chinese medicine, BV has been used as a non-steroidal anti-inflammatory drug to relieve pain and treat chronic inflammatory diseases, such as arthritis, rheumatism, and multiple sclerosis [18]. Pharmacological studies have demonstrated that BV possesses anti-mutagenic, anti-nociceptive, radioprotective, and anti-cancer activities [19,20,21,22,23]. In human melanoma cells, BV rapidly increased intracellular calcium (Ca^2+^) concentration and reactive oxygen species (ROS), and altered mitochondrial membrane potential, consequently led to the apoptotic cell death [24]. Cytosolic Ca^2+^ overload stimulates Ca^2+^-dependent catabolic enzymes (e.g., phospholipases, endonucleases, and proteases) and involves the several types of cell death including apoptosis, autophagy, necrosis, and anoikis [25]. MLT and PLA2, two major components of BV, have been also reported to induce apoptosis, necrosis, and cytolysis in various cancers via enhancement of calpain activity and Ca^2+^ entry, activation of death receptor signaling, and activation of caspases [26,27,28]. In addition, BV pharmaco-puncture and MLT are reportedly beneficial for the management of side effects associated with chemotherapy, such as peripheral neuropathy [29]. Recent studies investigating the beneficial effects of BV on stem cells have confirmed that BV promoted differentiation of umbilical cord-derived mesenchymal stem cells to osteocytes; moreover, the combination of BV and retinoic acid increased proliferation and differentiation of cholinergic neurons [30]. However, the effects of BV and its components on the selective elimination of iPSCs, as well as its ability to prevent teratoma formation, have not been examined.

In the present study, we investigated the cytotoxic activities of BV and its major components in undifferentiated iPSCs and iPSC-derived differentiated cells (iPSCs-Diff). Moreover, we elucidated the underlying mechanisms of cell death especially focused on the calcium-calpain pathway, and examined whether BV pre-treatment on iPSCs could prevent teratoma formation using an in ovo teratoma assay.

## 2. Results and Discussion

### 2.1. BV Exhibited Cytotoxicity in iPSCs but Not in iPSCs-Diff

**Figure 1:**
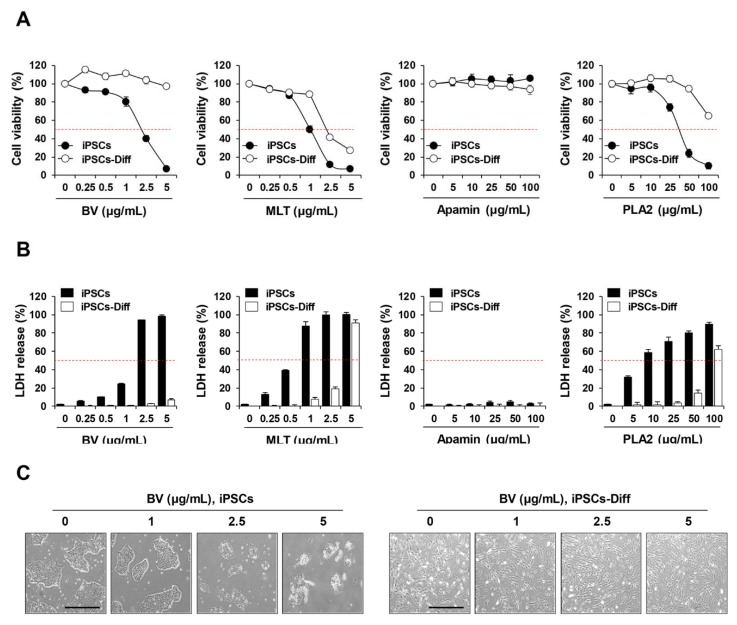
Effects of bee venom (BV), melittin (MLT), apamin, and phospholipase A2 (PLA2) on the cell viability and LDH release in iPSCs and iPSCs-Diff. (**A**) iPSCs and iPSCs-Diff were seeded on 24-well culture plates and then treated with the indicated concentrations of BV, MLT, apamin, and PLA2. After 24 h, viable cells were determined after crystal violet staining. Relative cell viability compared to untreated cells was calculated and expressed as means ± SD from triplicate samples. (**B**) iPSCs and iPSCs-Diff were treated with the indicated concentrations of BV, MLT, apamin, and PLA2 for 24 h and then LDH levels in culture supernatants were measured. The percentage LDH release calculated based on the background control and high control was expressed as means ± SD from triplicate samples. (**C**) iPSCs and iPSCs-Diff seeded in 12-well culture plates were incubated in the presence of BV at 1, 2.5, and 5 μg/mL. At 24 h post-treatment, cells were photographed under an inverted microscope. Scale bar = 100 μm.

To assess the cytotoxic effects of BV and its components on iPSCs and iPSCs-Diff, cells were treated with various concentrations of BV (0–5 µg/mL), MLT (0–5 µg/mL), apamin (0–100 µg/mL), and PLA2 (0–100 µg/mL) for 24 h; viability and cytotoxicity were determined by crystal violet staining and lactate dehydrogenase (LDH) release assay, respectively. As shown in Figure 1A, the cell viability of iPSCs was significantly reduced by BV (half maximal inhibitory concentration (IC50) = 2.34 µg/mL), MLT (IC50 = 0.93 µg/mL), and PLA2 (IC50 = 25.41 µg/mL) in a dose-dependent manner; no effect was observed for apamin. For iPSCs-Diff, cell viability was remarkably reduced by MLT (IC50 =1.98 µg/mL) and slightly reduced by PLA2; however, apamin exhibited no cytotoxic activity. In contrast, BV was non-toxic to iPSCs-Diff at concentrations up to 5 µg/mL, indicating that only BV was selectively cytotoxic to iPSCs. LDH release in iPSCs was greatly increased by treatment with BV, MLT, and PLA2 in a dose-dependent manner; while apamin had little effect. In iPSCs-Diff, similar elevations in LDH release were observed in response to MLT and PLA2, but not BV or apamin (Figure 1B). Morphological observation revealed that BV caused severe disruption of membrane integrity at 5 µg/mL and dramatically reduced cell viability; in iPSCs-Diff, cell appearance and viability were unaffected (Figure 1C). These data strongly indicate that BV confers selective cytotoxicity to undifferentiated iPSCs, but not to iPSCs-derived differentiated cells.

Due to the similarity in the growth rates of iPSCs and cancer cells, several anti-cancer drugs have been demonstrated to induce anti-proliferative and cytotoxic effects in iPSCs, while they were less effective for iPSCs-derived differentiated cells [31,32]. In previous studies, BV and its major components, including MLT and PLA2, have shown to confer various anti-cancer activities via the induction of apoptosis, necrosis, and cell lysis, combined with the inhibition of cell proliferation, angiogenesis, invasion, and metastasis [22,33]. Several studies have shown that tumor cells are more sensitive to the cytotoxic effects of BV and MLT, compared to non-tumor controls [34]. In addition, MLT has shown greater cytotoxicity to both tumor and non-tumor cells, relative to BV [35]. Similarly, in our study, MLT was cytotoxic both to iPSCs and iPSCs-Diff, therefore, subsequent experiments were carried out using only BV.

### 2.2. BV Rapidly Reduced Cell Membrane Integrity, F-Actin Expression, and Focal Adhesion-Related Proteins in iPSCs

**Figure 2:**
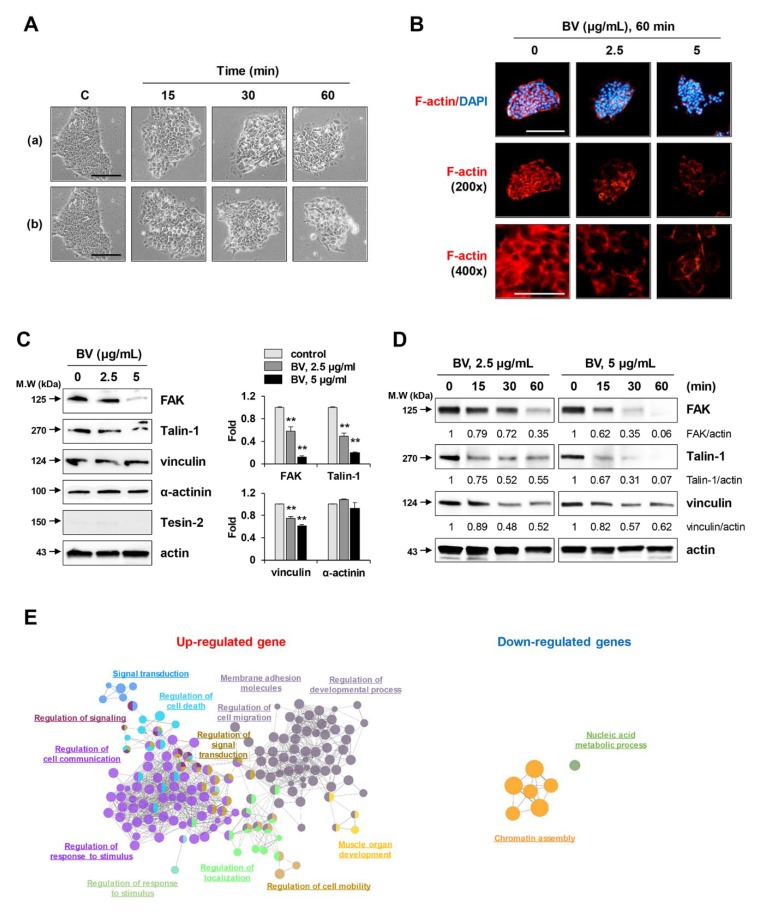
Effects of BV on the morphological change and F-actin intensity in iPSCs. (**A**) iPSCs were treated with 2.5 μg/mL (a) and 5 μg/mL (b) of BV and the morphological changes at 15, 30, and 60 min post-treatment were observed under an inverted microscope. Scale bar = 50 μm. (**B**) iPSCs seeded on the confocal dishes were treated with 2.5 and 5 μg/mL BV for 60 min and stained for F-actin with TRITC-conjugated phalloidin. Nuclei were counterstained with DAPI and then observed under a fluorescence microscope at low (200×) and high (400×) magnification. Scale bar = 50 μm. (**C**) iPSCs were treated with 2.5 and 5 μg/mL BV for 1 h and the levels of focal adhesion proteins were determined by Western blotting. Relative band intensities compared with BV-untreated iPSCs were calculated using Image J software after normalization to actin expression and expressed as means ± SD from two independent samples. **p < 0.01 vs. BV-untreated control (**D**) iPSCs were treated with 2.5 and 5 μg/mL BV. Protein samples at 15, 30, and 60 min post-treatment were harvested and then subjected to Western blotting. Data are representative of two independent experiments. (**E**) The enriched GO terms associated with DEGs were clustered (false discovery rate; FDR < 0.01) in network and represented with the same color. Representative functional terms for each cluster are shown. The size of each node indicates the enrichment significance of the GO term.

To investigate the mechanisms underlying BV-induced cell death, the morphological changes in BV-treated iPSCs were monitored by phase contrast microscopy. Untreated control iPSCs exhibited a compact shape. In contrast, BV treatment weakened the interactions with neighboring iPSCs; it then induced shrinkage, rounding of the cells, membrane blebbing, and release of cellular contents, all in a dose-dependent manner (Figure 2A). Interestingly, these morphological changes occurred rapidly, within approximately 15 min after BV treatment, and are therefore presumably critical events related to cell death. Adhesion to extracellular matrix and neighboring cells is reportedly necessary for the survival and maintenance of iPSC pluripotency [36]. In addition, it is widely accepted that the loss of focal contacts accompanied by degradation of the actin cytoskeleton leads to abrupt changes in the cell membrane (including cell rounding and membrane blebbing), which ultimately result in cell death [37,38]. Based on these observations, we examined changes in the F-actin cytoskeleton after staining with TRITC-conjugated phalloidin and determined the levels of focal adhesion-related proteins in BV-treated and untreated iPSCs. As shown in Figure 2B, F-actin filaments were evenly distributed in the cytoplasm and strongly expressed along the edges of untreated iPSCs. In contrast, significant disruption of F-actin filaments was evident in BV-treated iPSCs, as evidenced by shrinkage and clumping of F-actin bundles, which resulted in dramatic changes in cell morphology and adhesive capacity to Matrigel and adjacent cells.

Focal adhesion kinase (FAK) is overexpressed in numerous cancer types and plays important roles in the development of malignancy [39]; its effects include cell adhesion, migration, invasion, angiogenesis, proliferation, and survival. In human embryonic stem cells, integrin-associated FAK has been shown to support human embryonic stem cell survival, substrate adhesion, and maintenance of the undifferentiated state, while inhibition of FAK activity was shown to cause detachment-dependent apoptosis or differentiation [36,40]. In the process of cellular adhesion, focal adhesion-related proteins (e.g., FAK, talin, vinculin, paxillin, tensin, and actinine) are recruited to focal adhesions, where they become connected to the actin cytoskeleton [41]. Because we found that BV disrupted F-actin organization and reduced adhesion to Matrigel and adjacent cells, we examined the effects of BV on the expression of focal adhesion-associated proteins in iPSCs by Western blotting. As shown in Figure 2C, the levels of FAK, talin-1, and vinculin were all significantly reduced in a dose-dependent manner after treatment with BV for 1 h; there were no significant changes in the levels of α-actinin or tensin-2. Furthermore, FAK, talin-1, and vinculin all showed significant time-dependent reductions in protein levels from 15 min to 60 min after BV treatment (Figure 2D), consistent with the changes observed in cell morphology. Together, these data indicate that BV causes detachment and cell death via downregulation of focal adhesion in iPSCs.

The loss of cell membrane integrity in BV-treated iPSCs was also confirmed by measuring global gene expression changes using QuantSeq analysis. In first, time-dependently regulated genes were identified as differentially expressed genes (DEGs) in which 567 and 333 genes were upregulated and downregulated, respectively (Figure S1A). Then the biological functions associated with DEGs were presented as gene ontology (GO) network (Figure 2E) and GO treemap (Figure S1B). Time-dependently upregulated genes were associated with cell migration processes including cell mobility, cell communication, development, and membrane adhesion (FDR < 0.01). On the other hand, time-dependently downregulated genes were mainly associated with nucleosome assembly function. Taken together, BV induced rapid morphological changes in iPSCs and reduced nucleosome integrity by regulating the expression of various genes that could result in cell death.

### 2.3. BV Induced both Apoptosis and Necroptosis of iPSCs

**Figure 3:**
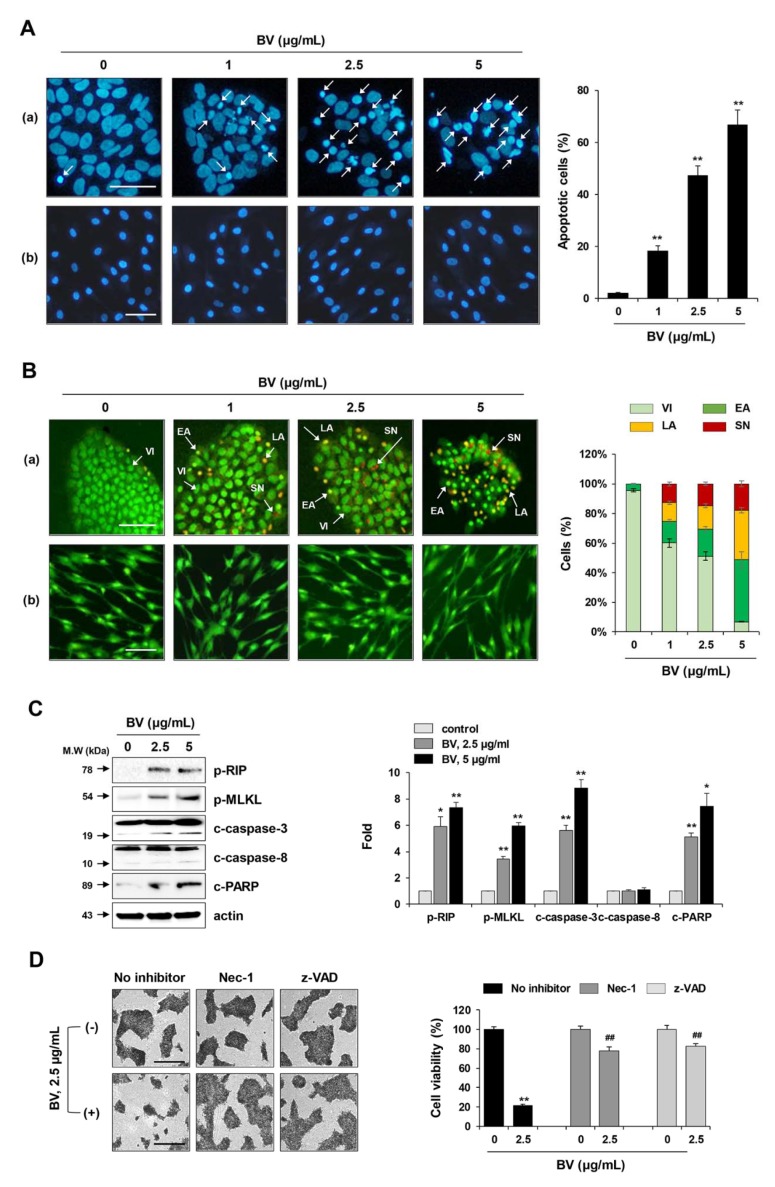
Induction of both apoptotic and necrotic cell death by BV treatment in iPSCs. (**A**) iPSCs (a) and iPSCs-Diff (b) were treated with 1, 2.5, and 5 μg/mL BV for 1 h, stained with DAPI, and then chromatin condensation was observed under a fluorescence microscope. Apoptotic cells (white arrow) in BV-treated or untreated iPSCs were counted and expressed as means ± SD from random 5 fields per sample. **p < 0.01 vs. BV-untreated control. Scale bar = 100 µm. (**B**) iPSCs (a) and iPSCs-Diff (b) were treated with 1, 2.5, and 5 μg/mL BV for 1 h, stained with AO and EB, and then observed under a fluorescence microscope. Viable cells (VI) were shown as green. Early apoptotic cells (EA) and late apoptotic cells (LA) were shown as condensed green color and yellow to orange color, respectively. Secondary necrotic cells (SN) were visible to red color. Each cell population was calculated and expressed as means ± SD from random 5 fields per sample. Scale bar = 100 µm. (**C**) iPSCs were treated with 2.5 and 5 μg/mL BV for 1 h and then subjected to Western blotting for measuring the levels of apoptosis- and necroptosis-related proteins. Relative band intensities compared with BV-untreated iPSCs were calculated using Image J software after normalization to actin expression and expressed as means ± SD from two independent samples. (**D**) iPSCs were pre-treated with or without Nec-1 (10 μM) and z-VAD (20 μM) for 30 min and then treated with 2.5 μg/mL BV. After 24 h, viable cells were measured after crystal violet staining. Relative cell viability compared with BV-untreated control iPSCs was expressed as means ± SD from triplicate samples. *p < 0.05 and **p < 0.01 vs. BV-untreated control, ^##^*p <* 0.01 vs. Nec-1 or z-VAD-untreated control. Scale bar = 100 µm.

To determine the mode of BV-induced cell death in iPSCs, BV-treated and untreated iPSCs were stained with DAPI (a cell-permeable DNA dye) and observed under a fluorescence microscope to assess morphological changes in the nucleus. As shown in Figure 3A, the nuclei of untreated iPSCs and iPSCs-Diff were normal with faint staining. In contrast, following treatment with BV at 1, 2.5, and 5 µg/mL for 1 h, typical features of apoptosis (e.g., nuclear condensation, increased intensity, and nuclear fragmentation) were observed in a dose-dependent manner in iPSCs (F = 194.3, *p* < 0.0001, one-way ANOVA), but not in iPSCs-Diff. Because rapid cell collapse was observed in response to BV treatment, we next examined whether BV induced necrotic cell death in iPSCs by acridine orange/ethidium bromide (AO/EB) staining, which can distinguish among healthy viable cells, early apoptotic cells, late apoptotic cells, and second necrotic cells. AO, a DNA binding dye that emits green fluorescence, can penetrate both live and dead cells. In contrast, EB is taken up only by dead cells, in which the cytoplasmic membrane integrity is disrupted, where it stains the nuclei red. When AO and EB are used together, healthy viable cells exhibit green fluorescence with normal morphology, early apoptotic cells exhibit green fluorescence with condensed nuclei, late apoptotic cells exhibit condensed yellow/orange fluorescence, and second necrotic cells exhibit condensed red fluorescence. As shown in Figure 3B, we found that BV treatment increased the proportions of early apoptotic cells, late apoptotic cells, and second necrotic cells in iPSCs in a dose-dependent manner. In contrast, apoptotic and necrotic cells were not observed in BV-treated iPSCs-Diff, indicating that BV selectively induced cell death in undifferentiated iPSCs, rather than in iPSCs-Diff.

Next, we investigated whether BV-induced necrotic cell death was caused by necrosis or necroptosis. Unlike necrosis, necroptosis is a highly regulated form of necrotic cell death, which is dependent on the phosphorylation of RIP and MLKL. As shown in Figure 3C, BV significantly enhanced the phosphorylation of RIP (Ser166) and MLKL (Ser358) in iPSCs. Moreover, BV treatment significantly increased the levels of cleaved caspase-3 and cleaved PARP in iPSCs; notably, caspase-8, a key inhibitor of necroptosis, was unaffected. BV-induced cell death was significantly inhibited by pre-treatment with necroptosis inhibitor necrostatin (Nec-1; RIP1 inhibitor) or apoptosis inhibitor z-VAD-fmk (pan-caspase inhibitor), indicating that BV induced both apoptosis and necroptosis in iPSCs (Figure 3D).

### 2.4. BV Enhanced Calcium Influx, Calpain Activity, and Reactive Oxygen Species (ROS) Generation of iPSCs

**Figure 4:**
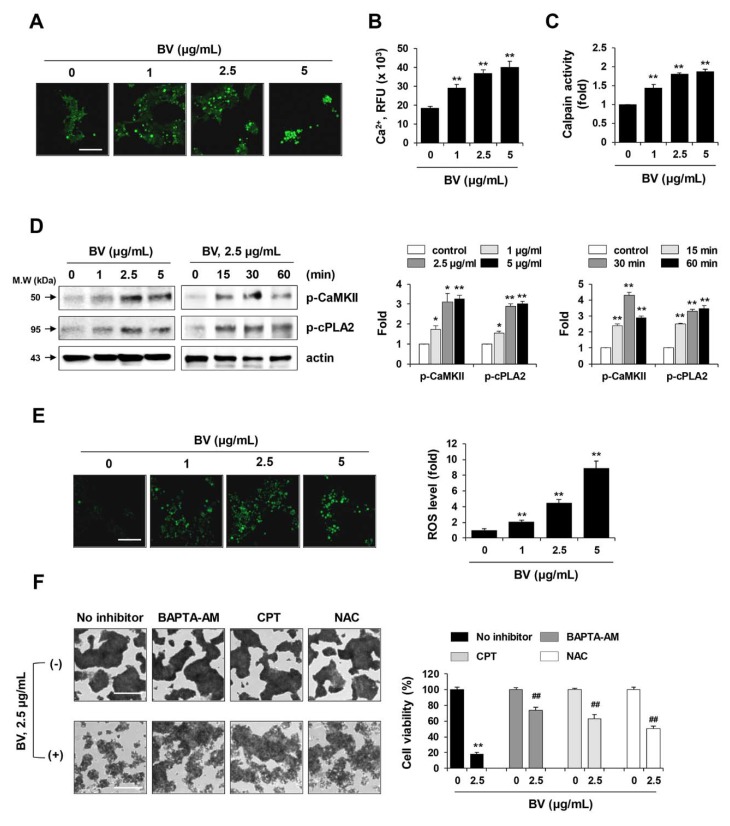
Effects of BV on the calcium response and reactive oxygen species (ROS) generation in iPSCs. (**A**) iPSCs cultured on confocal dishes were treated with 1, 2.5, and 5 μg/mL BV for 60 min. Cells were loaded with 2 μM Fura-2 AM, Ca^2+^ selective fluorescent indicator and then imaged on a fluorescence microscope. Scale bar = 100 µm. (**B**) After seeding iPSCs on the black wall/clear bottom 96-well plates, cells were treated with 1, 2.5, and 5 μg/mL BV for 60 min. Intracellular calcium was indicated by Fluo-8 green fluorescence and relative fluorescence unit (RFU) was expressed as means ± SD from triplicate samples. (**C**) BV-treated iPSCs were harvested and proteins were extracted. After adding calpain substrate Ac-LLY-AFC to the samples, calpain activity was monitored at Ex/Em = 400/505 nm. Relative calpain activity compared with BV-untreated iPSCs was expressed as means ± SD from triplicate samples. (**D**) Phosphorylation of CaMKII and cPLA2 in BV-treated iPSCs was determined by Western blotting. Relative band intensities compared with BV-untreated iPSCs were calculated using Image J software after normalization to actin expression. Data are expressed as means ± SD from two independent samples. (**E**) BV-treated iPSCs were stained with oxidative stress detection reagent and observed under a fluorescence microscope. Total ROS levels were measured by fluorescence reader (490/525 nm) and relative ROS levels compared with BV-untreated iPSCs were expressed as means ± SD from triplicate samples. Scale bar = 100 µm. (**F**) iPSCs were pre-treated with or without BAPTA-AM (1 μM), CPT (1 μM), and NAC (50 μM) for 30 min and then treated with 2.5 μg/mL BV for 24 h. After staining cells with crystal violet solution, relative cell viability compared with BV-untreated control iPSCs was expressed as means ± SD from triplicate samples. *p < 0.05 and **p < 0.01 vs. BV-untreated control, ^##^*p <* 0.01 vs. BAPTA-AM, CPT, or NAC-untreated control. Scale bar = 100 µm.

Elevated cytosolic Ca^2+^ triggers mitochondrial membrane permeability transition, increases ROS production, and induces the activation of Ca^2+^-dependent proteases (e.g., caspases and calpain) and Ca^2+^/calmodulin-dependent protein kinases (e.g., CaMKII), ultimately leading to apoptosis or necroptosis [25]. Calpain-mediated breakdown of focal adhesions and cytoskeletal proteins is responsible for cell rounding and membrane rupture during cell death [42]. To investigate the Ca^2+^ response in iPSCs following treatment with BV, cytosolic Ca^2+^ influx was measured using the Ca^2+^ selective indicator, Fura-2 AM. Under fluorescence microscopy, we observed that BV treatment increased the accumulation of cytosolic Ca^2+^ in a dose-dependent manner (Figure 4A). Quantitative measurement of intracellular Ca^2+^ using Fluo-8 AM, a green fluorescent Ca^2+^ binding dye, also showed that BV enhanced Ca^2+^ influx (F = 57.54, *p* < 0.0001, one-way ANOVA) (Figure 4B). Because Ca^2+^ is required for calpain activation, we next measured calpain activities in BV-treated iPSCs. As Ca^2+^ influx increased, the calpain activities in BV-treated iPSCs were significantly elevated in a dose-dependent manner (Figure 4C) (F = 110.3, *p* < 0.0001, one-way ANOVA). Previous analyses have shown that elevations in intracellular Ca^2+^ concentration can lead to the activation of CaMKII and cPLA2 [25]. Western blotting analyses showed that BV significantly increased the levels of phosphorylated CaMKII and cPLA2 in iPSCs, and these elevations appeared immediately after BV treatment (Figure 3D).

Finally, to assess the effects of BV on oxidative stress in iPSCs, we evaluated intracellular ROS levels. As shown in Figure 4E, green fluorescence intensity was elevated following treatment with 5 µg/mL BV, resulting in an approximately 9-fold increase in ROS production, compared with that of untreated iPSCs (F = 128.7, *p* < 0.0001, one-way ANOVA). Inhibitor assays showed that pre-treatment with BAPTA-AM (Ca^2+^ chelator), CPT (calpain inhibitor), and N-acetyl-L-cystein (NAC; ROS scavenger) prior to BV treatment significantly attenuated the reduction in cell viability of iPSCs, supporting the hypothesis that elevations in Ca^2+^ influx, calpain activation, and oxidative stress are critical events underlying BV-induced cell death in iPSCs.

### 2.5. BV Suppressed iPSC-Derived Teratoma Formation in Chick Chorioallantoic Membrane (CAM) Assay

**Figure 5:**
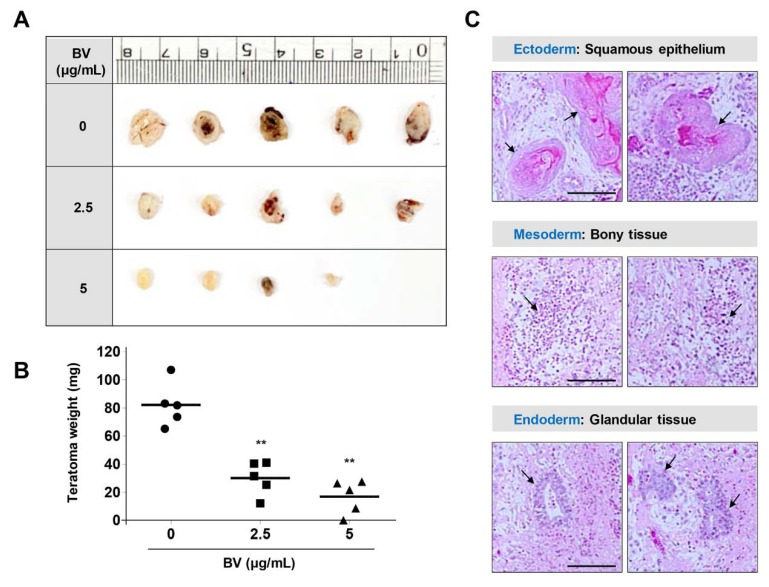
Suppression of in ovo teratoma formation of iPSCs by BV treatment. (**A**,**B**) On ED day 10, Matrigel plugs containing BV-treated or -untreated iPSCs were loaded on CAMs and then further grown for 8 days in an egg incubator. On ED day 18, teratomas were excised from chorioallantoic membrane (CAM), photographed, and measured for weight. Data are expressed as means ± SD (*n* = 5). **p < 0.01 vs. BV-untreated control (**C**) Teratomas generated from BV-untreated iPSCs were analyzed after hematoxylin-eosin (H&E) staining. Arrows indicate cells derived from three germ layers, including ectoderm, mesoderm, and endoderm, respectively.

To evaluate the limiting effects of BV on pluripotent differentiation capacity, we examined teratoma formation in iPSCs using a CAM assay. CAMs are essential for maintenance of the immunodeficient environment of the developing embryo, enabling grafted cells (e.g., both embryonic stem cells and cancer cells) to survive and proliferate without the risk of species-specific rejection [43,44]. In recent reports, CAM assays using fertilized eggs have been proposed as an alternative to xenograft assays using immunocompromised mice, which have proven undesirable due to a lack of standardization and the presence of various ethical concerns (e.g., animal welfare) [45]. To investigate whether BV could suppress teratoma formation, iPSCs were treated with 2.5 and 5 µg/mL BV for 24 h, suspended and solidified in Matrigel, and then loaded on CAMs of fertilized eggs at embryonic development (ED) day 10. On ED day 18, untreated iPSCs developed sizable teratomas (diameter, 5.1–8.5 mm; volume, 37.5–101.5 mm^3^), whereas BV-treated iPSCs developed relatively small teratomas of 2.5–24.8 mm^3^ and 0–8.1 mm^3^ at 2.5 and 5 µg/mL BV, respectively (Figure 5A). The mean teratoma weight of untreated iPSCs was 82.1 ± 15.7 mg, while the mean teratoma weights of iPSCs treated with 2.5 and 5 µg/mL BV were 30.0 ± 12.0 mg and 16.8 ± 12 mg, respectively; these results indicated that BV effectively suppressed iPSC-derived teratoma formation. Histological analysis showed that BV-untreated iPSCs developed teratomas containing three distinct germ layers (Figure 5C).

### 2.6. BV Exhibited no Genotoxicity in iPSC-Derived Differentiated Cells

**Figure 6:**
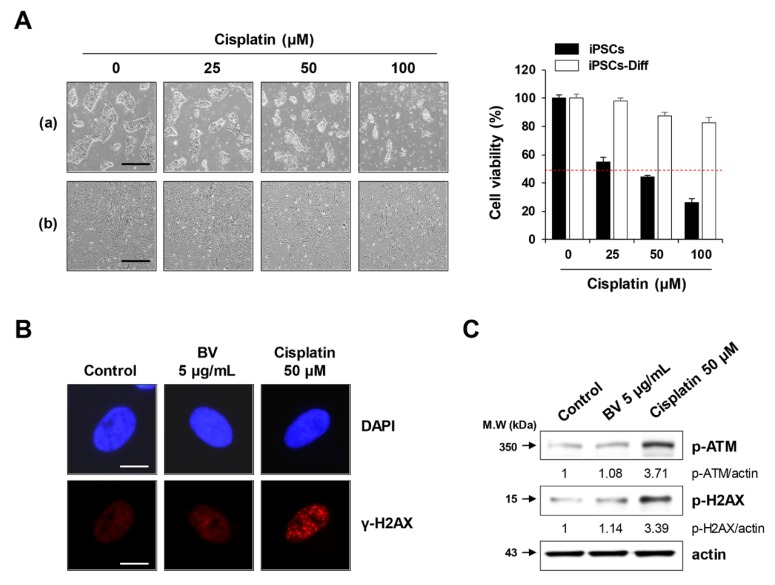
Effects of BV on the DNA damage in iPSCs-Diff. (**A**) iPSCs and iPSCs-Diff were seeded in 12-well culture plates and incubated in the presence of cisplatin at 25, 50, and 100 μM for 24 h. Cells were photographed under an inverted microscope and relative cell viability compared with cisplatin-untreated control cells was determined. Data are expressed as means ± SD from triplicate samples. Scale bar = 100 µm. (**B**) iPSCs-Diff grown on confocal dishes were treated with 5 μg/mL BV or 50 μM cisplatin for 24 h. Cells were stained with anti-p-H2AX antibody followed by Alexa 594-conjugated anti-rabbit antibody. After counterstaining nuclei with DAPI, γ-H2AX foci were observed under a fluorescence microscope. Scale bar = 20 µm. (**C**) BV- or cisplatin-treated iPSCs-Diff were analyzed for the protein levels of p-ATM and p-H2AX by Western blotting. Data are representative of two independent experiments.

Next, we examined whether BV was genotoxic to iPSCs-Diff. In general, DNA damaging agents, including anti-cancer drugs (i.e., cisplatin and doxorubicin) and γ-irradiation can cause DNA double-strand breaks and stimulate the phosphorylation of histone H2AX via ATM activation [46,47]. These breaks are visualized at the site of DNA damage as punctuate foci, regarded as γ-H2AX. Despite its potent anti-cancer activity, cisplatin is often hindered by serious side effects, including peripheral neurotoxicity, nephrotoxicity, and ototoxicity. In iPSC-derived cortical and peripheral neurons, cisplatin strongly inhibited neurite outgrowth [48]. In the present study, we observed that cisplatin significantly reduced the cell viability of iPSCs in a dose-dependent manner (F = 481.8, *p* < 0.0001, one-way ANOVA), whereas it showed minimal effects on iPSCs-Diff (Figure 6A). While cell viability was not significantly impacted, cisplatin treatment substantially increased the abundance of γ-H2AX foci in the nuclei of iPSCs-Diff and upregulated the protein levels of p-ATM and p-H2AX (Figure 6B,C), indicating that cisplatin exhibited genotoxicity in iPSCs-Diff. In contrast, γ-H2AX foci were generally absent in BV-treated iPSCs-Diff, with no significant elevations in p-ATM or p-H2AX levels compared to cisplatin-treated cells. These results indicated that BV was not genotoxic to iPSCs-Diff and was selectively cytotoxic to undifferentiated iPSCs.

### 2.7. BV Selectively Eliminated iPSCs co-Cultured with iPSC-Derived Differentiated Cells

**Figure 7:**
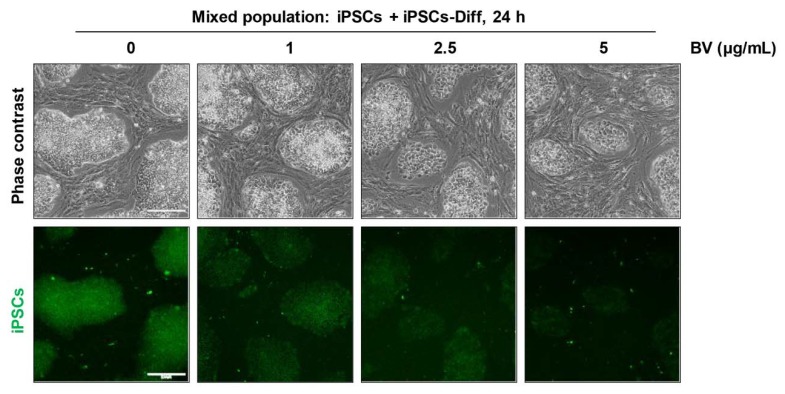
Selective elimination of iPSCs co-cultured with iPSCs-Diff. CellTracker green CMFDA dye-labeled iPSCs and un-labeled iPSCs-Diff were co-cultured, and treated with indicated concentrations of BV. After 24 h, cells were observed under an inverted microscope and a fluorescence microscope. Scale bar = 200 μm.

Finally, we examined whether BV selectively eliminated undifferentiated iPSCs when co-cultured with iPSCs-Diff. As shown in Figure 7, iPSCs were significantly decreased by treatment with BV in a dose-dependent manner, whereas iPSCs-Diff were retained. At 5 μg/mL BV, fluorescently-labeled iPSCs almost completely disappeared, indicating that BV can selectively cytotoxic to iPSCs but not to iPSCs-Diff even in the mixed cell population. These data strongly support that BV may be useful for obtaining teratoma-free cell sources for stem cell therapies by eliminating tumorigenic iPSCs. In recent studies, YM155 treatment during iPSCs-derived hepatocyte differentiation improved the quantity and quality of hepatocyte population, and prevented the potential risk of teratoma formation by selective removal of undifferentiated stem cells [49]. To verify the usefulness of BV as a potent anti-teratoma agent, we are currently investigating the beneficial effects of BV on iPSC-derived differentiated cells, including hepatocytes, dopaminergic neurons, and osteocytes.

## 3. Materials and Methods

### 3.1. Cell Culture

We used human induced pluripotent stem cells (iPSCs) which have been established from human foreskin fibroblasts (CRL-2429; American Tissue Culture Collection (ATCC), Manassas, VA, USA) in our laboratory [50]. iPSCs were maintained on mitomycin C-treated STO feeder cells (mouse embryo fibroblasts; CRL-1503; ATCC) or on hESC-qualified Matrigel Matrix (#354277; Corning, Bedford, MA, USA)-coated culture plates with mTeSR1 medium (Stem Cell Technologies, Vancouver, BC, Canada). iPSCs were gently detached by treatment with ReLeSR (Stem Cell Technologies). STO cells were cultured in Dulbecco’s modified Eagle’s medium (DMEM; Gibco, Grand Island, NY, USA) supplemented with 10% fetal bovine serum (FBS; Gibco), 100 Units/mL penicillin/100 μg/mL streptomycin (Gibco), 0.1 mM β-mercaptoethanol (β-ME; Gibco), and 1% non-essential amino acid (NEAA; Gibco). iPSCs-derived differentiated cells (iPSCs-Diff) in this study were generated as described previously with slight modification [51]. iPSCs-Diff showed fibroblast-like morphology and replicated in DMEM containing 10% FBS, 100 Units/mL penicillin/100 μg/mL streptomycin, 0.1 mM β-ME, and 1% NEAA. iPSCs-Diff were characterized that pluripotency marker genes including *OCT4*, *NANOG*, and *DNMT3B* were dramatically diminished, whereas mesenchymal progenitor cell marker genes including *CD29*, *CD44*, *CD73*, and *CD105* were remarkably increased compared to parent iPSCs (Figure S2A).

### 3.2. Chemicals

Purified BV was purchased from Chung-Jin Biotech (Asan, Korea), dissolved in phosphate-buffered saline (PBS), and aliquots were stored at −20 °C. MLT, apamin, and PLA2 from honeybee venom were all obtained from Sigma Chemical Co. (St Louis, MO, USA). Acridine orange (AO), ethidium bromide (EB), 4’,6-Diamidino-2-phenylindole (DAPI), necrostatin-1 (Nec-1), BAPTA-AM, calpeptin (CPT), z-VAD-fmk, and TRITC-labeled phalloidin from *Amanita phalloides* were purchased from Sigma Chemical Co. and N-acetyl-L-cysteine (NAC) was obtained from Calbiochem (San Diego, CA, USA).

### 3.3. Cell Viability and Cytotoxicity Assay

iPSCs and iPSCs-Diff were seeded on 12-well culture plates, incubated overnight, and then treated with increasing concentrations of BV (0–5 μg/mL), MLT (0–5 μg/mL), apamin (0–100 μg/mL), and PLA2 (0–100 μg/mL). After 24 h, culture supernatants were collected and measured for lactate dehydrogenase (LDH) activity using Cytotoxicity Detection Kit (Roche Diagnostics, Mannheim, Germany) according to the manufacturer’s protocol. To examine cell viability, treated cells were washed with PBS two times, and then stained with crystal violet solution (0.2% crystal violet in 20% methanol) for 30 min. After washing with distilled water thoroughly, cells were photographed and solubilized with 1% sodium dodecyl sulfate (SDS) solution. The absorbance was measured at 590 nm using a SpectraMax3 microplate reader (Molecular Devices, Sunnyvale, CA, USA). To examine selective elimination ability of BV, iPSCs cultured in mTeSR1 on Matrigel-coated 12-well culture plates were labeled with 20 μM CellTracker Green CMFDA dye (Thermo Scientific, Waltham, MA, USA) at 37 °C for 30 min. The residual fluorescence was washed out with PBS, and then un-labeled iPSCs-Diff were added and co-cultured with iPSCs in mTeSR1. After incubating in the presence or absence of BV, cells were observed under an inverted microscope and a fluorescence microscope.

### 3.4. Measurement of Apoptotic and Necrotic Cells by DAPI and AO/EB Staining

To investigate the BV-induced cell death, iPSCs and iPSCs-Diff grown on the 35-mm glass bottom dishes (SPL Life Sciences, Pocheon, Korea) were treated with indicated concentrations of BV for 1 h. To identify apoptotic bodies, cells were fixed with 10% neutral buffered formalin solution (Sigma Chemical Co.) for 30 min at room temperature (RT), stained with DAPI (1 μg/mL) for 20 min at RT, and then observed under an Olympus IX71 inverted fluorescence microscope. In addition, BV-treated or -untreated cells were incubated with a mixture of AO/EB (100 μg/mL/100 μg/mL in PBS) for 20 min at RT. Viable cells (VI: green), early apoptotic cells (EA: condensed green), late apoptotic cells (LA: yellow to orange), and secondary necrotic cells (SN: red) were observed under an Olympus IX71 inverted fluorescence microscope.

### 3.5. Immunofluorescence Analysis for F-Actin and γ-H2AX Foci

To visualize the F-actin, iPSCs grown on Matrigel-coated 35-mm glass bottom dishes were treated with BV for 1 h. After fixation, cells were stained with TRITC-labeled phalloidin (diluted to 1 μg/mL) for 1 h at RT, counterstained with DAPI for 20 min at RT, and then observed under an Olympus IX71 inverted fluorescence microscope. To detect γ-H2AX foci, iPSCs-Diff grown on 35-mm glass bottom dishes were treated with 5 μg/mL BV or 100 μM cisplatin for 24 h. After fixation, cells were treated with permeabilization buffer (0.1% Triton X-100 in PBS) for 30 min at RT, blocked with blocking buffer (3% FBS in PBS) for 1 h at RT, and then stained with anti-p-H2AX antibody (diluted 1:1000 in blocking buffer) overnight (O/N) at 4 °C, followed by Alexa Fluor 594 anti-rabbit IgG antibody (diluted 1:1000 in blocking buffer) at RT for 3 h. After counterstaining nuclei with DAPI for 20 min at RT, γ-H2AX foci were observed under an Olympus IX71 inverted fluorescence microscope.

### 3.6. Immunoblotting

Whole cell lysates were obtained using mammalian protein extraction reagent (M-PER; Thermo Scientific, Rockford, IL, USA) and their protein concentrations were calculated using a bicinchoninic acid (BCA) kit (Thermo Scientific). Proteins (20 μg/well) were resolved by SDS-PAGE, immunoblotted using specific primary antibodies (1:1000 dilution) and HRP-linked secondary antibodies (1:4000 dilution), and then visualized under an ImageQuant LAS 4000 mini (GE Healthcare, Piscataway, NJ, USA) using a Clarity Western ECL substrate (Bio-Rad, Hercules, CA, USA). Primary antibodies against FAK (#3285), talin-1 (#4021), vinculin (#4650), α-actinin (#6487), tensin-2 (#11990), p-RIP (#65746), p-MLKL (#91689), caspase-3 (#9662), caspase-8 (#9746), PARP (#9542), p-CaMKII (#12716), p-cPLA2 (#2831), p-ATM (#5883), and p-H2AX (#2577) were purchased from Cell Signaling Technology (Danvers, MA, USA). Anti-actin antibody (sc-47778) was obtained from Santa Cruz Biotechnology Inc. (Santa Cruz, CA, USA). Secondary antibodies, HRP-linked anti-rabbit antibody (#7074) and anti-mouse antibody (#7076), were purchased from Cell Signaling Technology.

### 3.7. Intracellular Calcium (Ca^2+^) Measurement

To image cytosolic free Ca^2+^, iPSCs were seeded on Matrigel-coated 35-mm glass bottom dishes and treated with indicated concentrations of BV for 1 h. After washing with pre-warmed Hank’s balanced salt solution (HBSS, Gibco, containing Ca^2+^ and Mg^2+^, no phenol red), cells were incubated with fluorescent Ca^2+^ selective indicator, Fura-2 AM (2 μM diluted in HBSS) for 30 min at RT in the dark. After washing with pre-warmed HBSS, cells were observed under Olympus IX71 inverted fluorescence microscope. In addition, the intracellular Ca^2+^ flux was quantified in BV-treated and -untreated iPSCs using Fluo-8 Calcium Flux Assay Kit (Abcam, Cambridge, MA, USA) according to the manufacturer’s instruction. In brief, iPSCs were cultured O/N in a Matrigel-coated 96-well black wall/clear bottom plate (Costar, Cambridge, MA, USA) and treated with indicated concentrations of BV for 1 h. The culture medium was removed and then 100 μL Fluo-8 dye loading solution was added into the cells. After incubation at 37 °C for 1 h, fluorescence intensity was monitored at Ex/Em = 490/525 nm using a SpectraMax3 microplate reader and Ca^2+^ flux was expressed as relative fluorescence units (RFU).

### 3.8. Calpain Activity Assay

iPSCs were treated with indicated concentrations of BV for 1 h and then calpain activity was measured using Calpain Activity Assay Kit (Fluorometric; Abcam) according to the standard protocol. In brief, BV-treated and -untreated iPSCs were harvested, washed with cold PBS, and then resuspended in extraction buffer. After collecting supernatants by centrifugation, protein concentration was determined using a BCA kit. Cell lysate (100 μg in 85 μL extraction buffer) was mixed with 10 μL of 10 × reaction buffer and 10 μL calpain substrate Ac-LLY-AFC. After further incubation for 1 h at 37 °C in the dark, fluorescence was measured at Ex/Em = 400/505 nm using a SpectraMax3 microplate reader.

### 3.9. Detection of Intracellular Reactive Oxygen Species (ROS)

Cells grown on Matrigel-coated 35-mm glass bottom dishes were treated with indicated concentrations of BV for 1 h and then intracellular ROS generation was detected using ROS-ID Total ROS detection kit (Enzo Life Sciences, Farmingdale, NY, USA) according to the manufacturer’s instruction. In brief, oxidative stress detection reagent was added into BV-treated and -untreated iPSCs and the green fluorescent images were obtained using fluorescence microscope. In addition, fluorescence intensities were measured at Ex/Em = 490/525 nm using a SpectraMax3 microplate reader.

### 3.10. RNA Isolation, Library Preparation, and Sequencing for QuantSeq Analysis

Total RNAs from BV-untreated and –treated iPSCs were extracted using the RNeasy Mini Kit (Qiagen, Valencia, CA, USA) according to the manufacturer’s instructions. After quality assessment of the RNA by 2100 Bioanalyzer Instrument (Agilent, Santa Clara, CA, USA), only samples with an RNA integrity number >7.0 were included in the QuantSeq analysis. From 500 ng total RNA, double-stranded cDNA libraries were prepared using a TruSeq Stranded Total RNA LT Sample Prep Kit with Rico-zero Gold (Illumina, San Diego, CA, USA) and then RNA sequencing was performed using a NovaSeq 6000 Sequencing system (Illumina). For QuanSeq data analysis, QuantSeq reads were aligned to a reference genome (genome assembly version of hg38) using Bowtie2 to estimate the number of transcripts [52]. Read count data were then normalized to obtain fragments per kilobase of million reads mapped (FPKM) using the edgeR R package [53]. Genes with expression ratios above 2 fold or below 0.5 compared with control samples were considered differentially expressed genes (DEGs). Functional network of gene ontology (GO) terms was constructed by querying DEGs from each experimental condition into the ClueGO Cytoscape plugin program under the default parameter settings including false discovery rate (FDR) < 0.01 [54]. The GO term treemap was created by implementing the ReviGO algorithm [55], which removes the GO term redundancy. Original enriched GO terms (adjusted *p*-values < 0.001) were obtained from DAVID resources using DEGs [56]. Raw datasets can be accessed at the Sequence Read Archives (SRA; http://www.ncbi.nlm.nih.gov/sra) under the accession number GSE148378. Quantitative real-time PCR was performed as described previously [57] using specific primers (Figure S2B).

### 3.11. In Ovo Teratoma Formation Assay

To investigate in ovo teratoma formation of iPSCs, we used fertilized chicken eggs obtained from Pulmuone Co., Ltd. (Seoul, Korea). After setting the first day of experiment as embryonic development (ED) day 0, eggs were incubated at 37 °C with 65% humidity in egg incubators (MX-190 CD; R-COM, Gimhae, Korea). On ED day 3, 5 mL albumin was carefully removed using a syringe and sealed by paraffin. Round window was made at the blunt end of eggs and eggs were incubated after covering windows with transparent adhesive tape. On ED day 10, iPSCs pre-treated with or without 2.5 and 5 μg/mL BV for 24 h were mixed with 50 μL cold Matrigel and then loaded on the CAMs. After resealing the windows, eggs were further incubated in egg incubator for 8 days. On ED day 18, teratomas developed on CAMs were excised, photographed, and weighed. Histological analysis of teratomas was conducted as described previously [57]. In brief, teratomas fixed with 10% neutral buffered formalin solution were paraffin-embedded using an automated tissue processor (Shandon Citadel 2000; Thermo Scientific). After cutting paraffin blocks to 3–4 μm in thickness using an automated microtome (RM2255; Leica Biosystems, Nussloch, Germany), sections were hematoxylin-eosin (H-E) stained and analyzed for three different embryonic germ layers under a light microscope (Eclipse 80*i*; Nikon, Tokyo, Japan).

### 3.12. Statistical Analysis

Data are presented as means ± standard deviation (SD). Statistical significance between two groups was analyzed with Student’s *t*-test and treatment efficiency was analyzed with one-way analysis of variance (ANOVA) followed by Dunnett’s test. All variables were analyzed using GraphPad Prism Software (GraphPad Software, Inc., La Jolla, CA, USA). The value of *p <* 0.05 was taken to indicate a significant difference.

## 4. Conclusions

This is the first study to demonstrate the beneficial effects of BV in iPSCs with respect to inhibition of teratoma formation. We showed that BV rapidly induced membrane degradation and loss of focal adhesion in iPSCs, resulting in both apoptotic and necroptotic cell death. We also showed that elevations in cytosolic Ca^2+^, calpain activity, and ROS generation were closely associated with BV-induced cell death in iPSCs. Moreover, BV efficiently suppressed the in ovo growth of teratomas. We found that BV was neither cytotoxic nor genotoxic to iPSCs-Diff, while MLT and PLA2 caused considerable cytotoxic effects in iPSCs-Diff. Together, these data provide evidence that BV is an effective and safe agent for the preparation of cells for stem cell-based therapy. Further investigations will be necessary to assess the anti-teratoma efficacy of BV during in vitro differentiation to specific cell types.

## Supplementary Materials

Supplementary materials can be found at https://www.mdpi.com/1422-0067/21/9/3265/s1.

## Abbreviations

iPSCs	induced pluripotent stem cells
BV	bee venom
PLA2	phospholipase A2
FAK	focal adhesion kinase
DEGs	differentially expressed genes
GO	gene ontology
DAPI	4’,6-Diamidino-2-phenylindole
AO	acridine orange
EB	ethidium bromide
Nec-1	necrostatin
ROS	reactive oxygen species
CPT	calpeptin
NAC	N-acetyl-L-cysteine
CAM	chorioallantoic membrane
ED	embryonic development
