# Supplementary material for: Elimination of Teratogenic Human Induced Pluripotent Stem Cells by Bee Venom via Calcium-Calpain Pathway
Source: Int J Mol Sci. 2020 May 5;21(9):3265. doi: 10.3390/ijms21093265 (PMC7246707; doi:10.3390/ijms21093265)
Supplement: Supplementary file 1 [file ijms-21-03265-s001.pdf]

Supplementary Figure 1

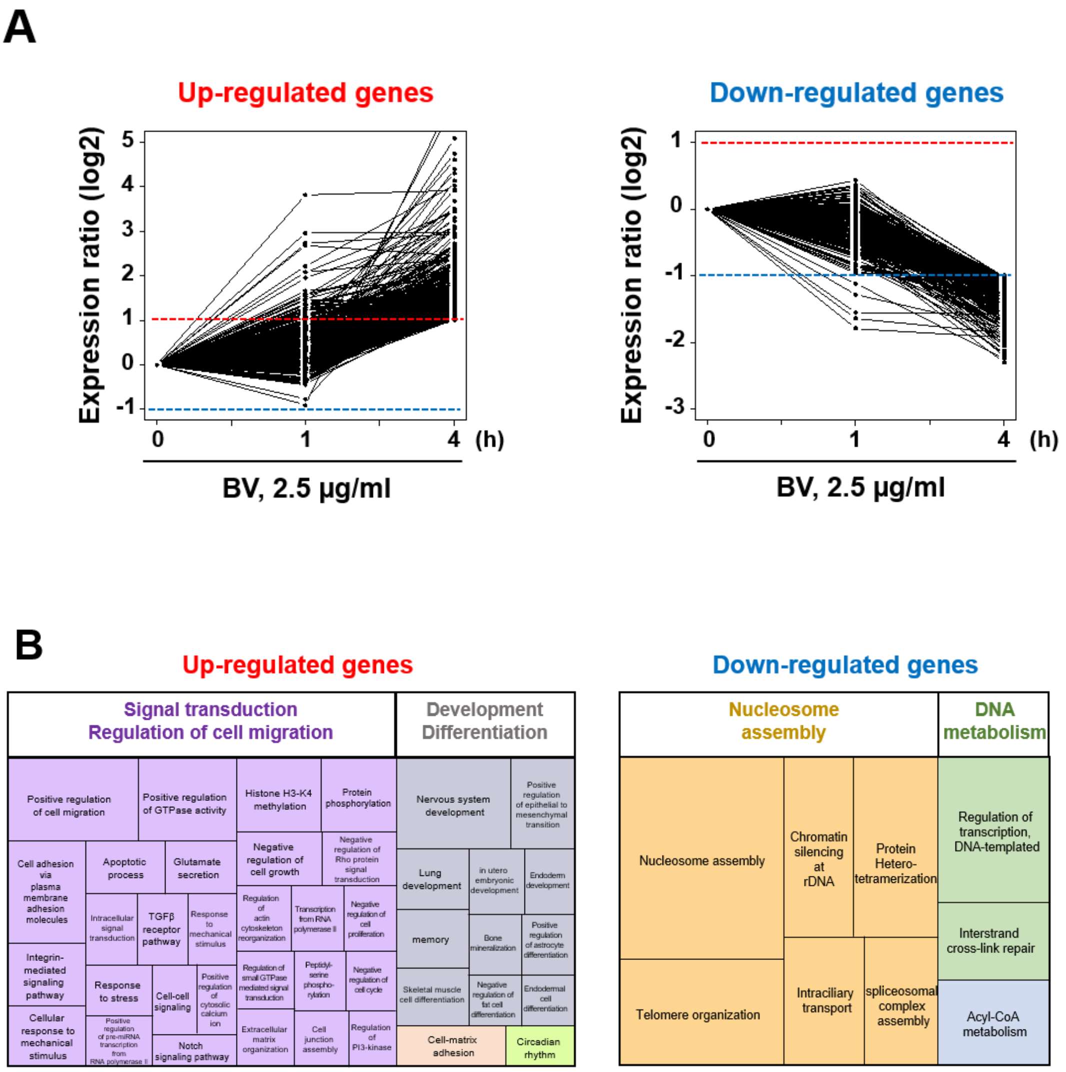

Supplementary Figure 1. QuentSeq analysis of BV-treated iPSCs. **(A)** iPSCs were treated with 2.5 μg/ml BV for 1 and 4 h. After measurement of expression level of genes using QuantSeq analysis, time-dependently up-regulated 567 genes and time-dependent down-regulated 333 genes were identified as DEGs. Threshold line for 2 or 0.5-fold ratio was shown in red or blue color, respectively. **(B)** Redundancy removed GO terms associated with DEGS were presented as treemap. Functionally related GO terms were represented with the same color. The area is proportional to the statistical significance of each GO term.

Supplementary Figure 2

A

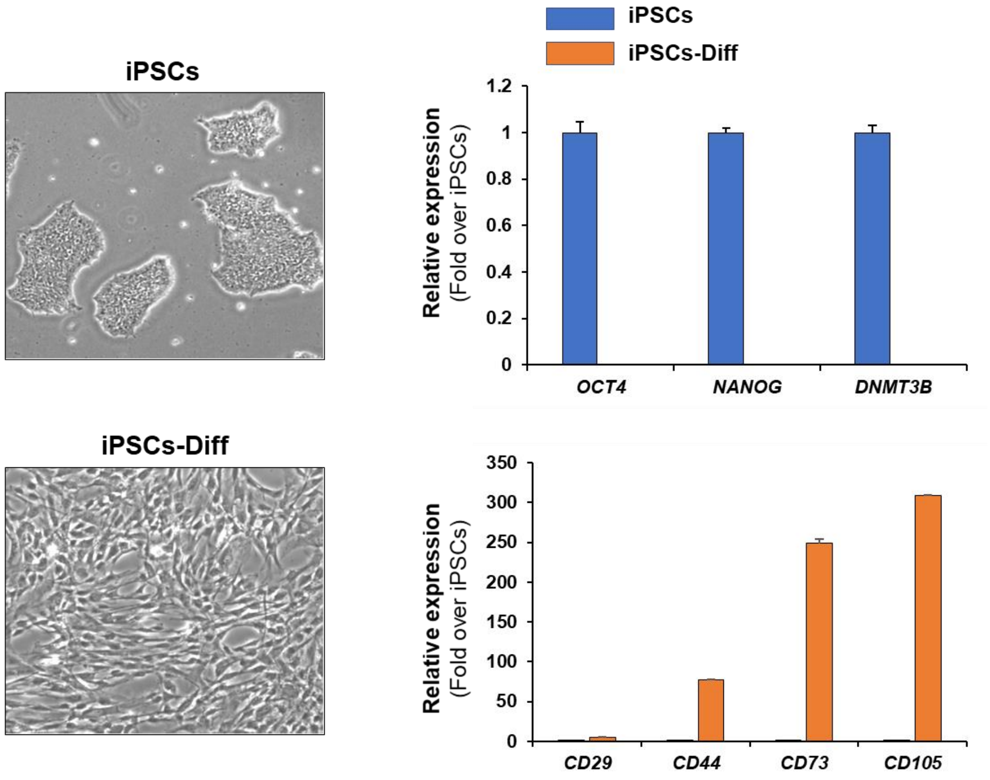

B

Gene	Forward primer (5´– 3´)	Reverse primer (5´– 3´)
OCT4	GGGAGGAGCTAGGGAAAGAAAA	ATTGAACTTCACCTTCCCTCCA
NANOG	TTAATAACCTTGGCTGCCGTCT	AATAAGCAAAGCCTCCCAATCC
DNMT3B	TCTCACGGTTCCTGGAGTGTAA	GTAGGTTGCCCCAGAAGTATCG
CD29	GTAACCAACCGTAGCAAAGGA	TCCCCTGATCTTAATCGCAAAAC
CD105	TGCACTTGGCCTACAATTCCA	AGCTGCCCACTCAAGGATCT
CD73	GCCTGGGAGCTTACGATTTTG	TAGTGCCCTGGTACTGGTCG
CD44	CTGCCGCTTTGCAGGTGTA	CATTGTGGGCAAGGTGCTATT
GAPDH	CCTCAACGACCACTTTGTCAAG	TCTTCCTCTTGTGCTCTTGCTG

Supplementary Figure 2. Characterization of iPSCs-derived differentiated cells (iPSCs-Diff). **(A)** Cell morphology of iPSCs and iPSCs-Diff was observed under an inverted microscope. Expression levels of pluripotent markers (e.g., OCT4, NANOG, DNMT3B) and mesenchymal progenitor cell markers (e.g., CD29, CD44, CD73, CD105) in iPSCs and iPSCs-Diff were analyzed by qPCR using specific primers **(B)**.
